# Supplementary material for: Role of community and sexual contacts as drivers of MPXV clade I
Source: Nat Health. 2026 Apr 6;1(6):595–606. doi: 10.1038/s44360-026-00080-7 (PMC13246439; doi:10.1038/s44360-026-00080-7)
Supplement: Supplementary file 3 — Peer Review File [file 44360_2026_80_MOESM3_ESM.pdf]

nature portfolio

Peer Review File

The Role of Community and Sexual Contacts as Drivers of Clade I Mpox Outbreaks

Corresponding Author: Dr Akira Endo

This manuscript has been previously reviewed at another journal. This document only contains information relating to versions considered at Nature Health.

This file contains all reviewer reports in order by version, followed by all author rebuttals in order by version.

Version 0:

Reviewer comments:

Reviewer #1

(Remarks to the Author)
I was asked to check whether the revisions to the comments of R3 were adequate, since R3 was no longer available. I read the revised paper and the rebuttal portion for R3's comments. I think the author responses to Comments 2 and 3 were adequate, but the response to Comment 1 does not satisfy the original reviewer's comment.

Comment 1: validity of simplified model

The author's clarification about 1% versus 2% sexually active females is noted, although the original reviewer mentioned that this was just one of many other parameter choices that must be made arbitrarily when constructing a network model. I agree with the reviewer's concern that the network models have too many degrees of freedom, and that it is trivial to make them replicate the results using the NGM model. Tweaking the POC network models to match the NGM predictions of %sexual does not convince me that the NGM predictions are valid, and I don't think the original reviewer would have been convinced either.

The following response regarding using a 'unit of sexual contact' may address that specific concern, and the response about the absolute values of R0 is fine, except the authors note that they must require a well-connected network to allow use of mean-field theory, which makes the response to the earlier problem about the POC networks weaker.

Comment 2: validity of R projections

The response about figure 2B is fine.

The response about spatial aggregation acknowledges some issues it may create, but does not seem to address the reviewer concern. However, the reviewer seems like they would accept a statement of caveats, which the authors provided in the revision.

The authors mention the limitation about biases due to age-specific underascertainment.

Comment 3:

Authors' clarification seems adequate.

(Remarks on code availability)

Open Access This Peer Review File is licensed under a Creative Commons Attribution 4.0 International License, which permits use, sharing, adaptation, distribution and reproduction in any medium or format, as long as you give appropriate credit to the original author(s) and the source, provide a link to the Creative Commons license, and indicate if changes were made.
In cases where reviewers are anonymous, credit should be given to 'Anonymous Referee' and the source.
The images or other third party material in this Peer Review File are included in the article's Creative Commons license, unless indicated otherwise in a credit line to the material. If material is not included in the article's Creative Commons license and your intended use is not permitted by statutory regulation or exceeds the permitted use, you will need to obtain permission directly from the copyright holder.
To view a copy of this license, visit https://creativecommons.org/licenses/by/4.0/

26 December 2025

Dear Editor,

Thank you for your encouraging assessment and detailed instructions to finalise our manuscript. As requested, we have formatted our manuscript to comply with the journal policy and included point-by-point responses to the comments. Regarding the guideline on sex and gender, our study has already disaggregated sex in our analysis, and the guidance on the reporting of sex was not applicable to our study since we only used publicly available data.

Thank you once again for handling our manuscript.

Yours sincerely,
Akira Endo on behalf of all authors.

—-------

Point-by-point responses

Reviewers' Comments:

Reviewer #1 (Remarks to the Author):

I was asked to check whether the revisions to the comments of R3 were adequate, since R3 was no longer available. I read the revised paper and the rebuttal portion for R3's comments. I think the author responses to Comments 2 and 3 were adequate, but the response to Comment 1 does not satisfy the original reviewer's comment.

> We appreciate the reviewer's time and effort to assess our responses to the original reviewer.

Comment 1: validity of simplified model

The author's clarification about 1% versus 2% sexually active females is noted, although the original reviewer mentioned that this was just one of many other parameter choices that must be made arbitrarily when constructing a network model. I agree with the reviewer's concern that the network models have too many degrees of freedom, and that it is trivial to make them replicate the results using the NGM model. Tweaking the POC network models to match the NGM predictions of %sexual does not convince me that the NGM predictions are valid, and I don't think the original reviewer would have been convinced either.

> We thank the reviewer for raising this concern. We totally agree with the reviewer's view that tweaking the POC network models to replicate the similar NGM predictions of %sexual to the main analysis would not serve as a validation. As we stated in the previous response, our POC analysis was not intended to argue this point. Instead, we aimed to show that the NGM approach (i.e. approximation of the full network by the next generation matrix) can yield almost the same %sexual value as that from the adjacency matrix (i.e. containing the full individual-level network information). The %sexual value from the adjacency matrix can be regarded as the ground-truth value of the POC network, not the network in the main analysis, and we attempted to show that our NGM approach can reliably recover this ground-truth. To this end, we generated two example POC networks, whose characteristics roughly (if not completely) mimic the network in the main analysis. In this light, the resemblance between our POC networks and the results in the main analysis are only for contextual relevance and not validation in itself. Just because our

POC networks are different from the networks in the main analysis in many aspects does not affect our main argument here—that our NGM approach can reasonably approximate the underlying network structure.

We have edited our explanation for further clarity in this regard:

SI, “Proof of concept using an individual-level network model” section:
… The relative contribution of sexual contacts to R0 for networks 1 and 2, measured as the relative reduction in the dominant eigenvalue of adjacency matrix when all sexual contact links are removed, was ensured to generally align with the range of our estimates for clade Ib in the main analysis through our choices of network parameters (Table S2).

The following response regarding using a ‘unit of sexual contact’ may address that specific concern, and the response about the absolute values of R0 is fine, except the authors note that they must require a well-connected network to allow use of mean-field theory, which makes the response to the earlier problem about the POC networks weaker.

> We thank the reviewer for this comment. We acknowledge that the requirement for (almost) well-connected networks is a key caveat, although we believe the use of mean-field theory through this assumption in STI modelling is a common practice (https://onlinelibrary.wiley.com/doi/10.1155/2011/284909). Given that the scope of this paper is not to provide a methodological framework applicable to any form of sexual contact networks, we believe the stated caveat would be sufficient for the readers to assess the potential limitation in our approach.

Comment 2: validity of R projections

The response about figure 2B is fine.

The response about spatial aggregation acknowledges some issues it may create, but does not seem to address the reviewer concern. However, the reviewer seems like they would accept a statement of caveats, which the authors provided in the revision.

The authors mention the limitation about biases due to age-specific underascertainment.

> We thank the reviewer for this assessment.

Comment 3:

Authors’ clarification seems adequate.

> We thank the reviewer for this assessment.
